# Novel CaF_2_ Nanocomposites with Antibacterial Function and Fluoride and Calcium Ion Release to Inhibit Oral Biofilm and Protect Teeth

**DOI:** 10.3390/jfb11030056

**Published:** 2020-08-01

**Authors:** Heba Mitwalli, Abdulrahman A. Balhaddad, Rashed AlSahafi, Thomas W. Oates, Mary Anne S. Melo, Hockin H. K. Xu, Michael D. Weir

**Affiliations:** 1 Program in Dental Biomedical Sciences, University of Maryland School of Dentistry, Baltimore, MD 21201, USA; hmitwalli@umaryland.edu (H.M.); aabalhaddad@umaryland.edu (A.A.B.); rashed.alsahafi@umaryland.edu (R.A.); 2 Department of Restorative Dental Science, College of Dentistry, King Saud University, Riyadh 11451, Saudi Arabia; 3 Department of Restorative Dental Sciences, College of Dentistry, Imam Abdulrahman bin Faisal University, Dammam 31441, Saudi Arabia; 4 Department of Restorative Dental Sciences, College of Dentistry, Umm Al-Qura University, Makkah 24211, Saudi Arabia; 5 Department of Advanced Oral Sciences and Therapeutics, School of Dentistry, University of Maryland, Baltimore, MD 21201, USA; TOates@umaryland.edu (T.W.O.); MMelo@umaryland.edu (M.A.S.M.); HXu2@umaryland.edu (H.H.K.X.); 6 Division of Operative Dentistry, Department of General Dentistry, University of Maryland School of Dentistry, Baltimore, MD 21201, USA; 7 Center for Stem Cell Biology & Regenerative Medicine, University of Maryland School of Medicine, Baltimore, MD 21201, USA; 8 Marlene and Stewart Greenebaum Cancer Center, University of Maryland School of Medicine, Baltimore, MD 21201, USA

**Keywords:** dental nanocomposite, calcium fluoride nanoparticles, remineralization, antibacterial, protein repellent, oral biofilm

## Abstract

(1) Background: The objective of this study was to develop a novel dental nanocomposite containing dimethylaminohexadecyl methacrylate (DMAHDM), 2-methacryloyloxyethyl phosphorylcholine (MPC), and nanoparticles of calcium fluoride (nCaF_2_) for preventing recurrent caries via antibacterial, protein repellent and fluoride releasing capabilities. (2) Methods: Composites were made by adding 3% MPC, 3% DMAHDM and 15% nCaF_2_ into bisphenol A glycidyl dimethacrylate (Bis-GMA) and triethylene glycol dimethacrylate (TEGDMA) (denoted BT). Calcium and fluoride ion releases were evaluated. Biofilms of human saliva were assessed. (3) Results: nCaF_2_+DMAHDM+MPC composite had the lowest biofilm colony forming units (CFU) and the greatest ion release; however, its mechanical properties were lower than commercial control composite (*p* < 0.05). nCaF_2_+DMAHDM composite had similarly potent biofilm reduction, with mechanical properties matching commercial control composite (*p* > 0.05). Fluoride and calcium ion releases from nCaF_2_+DMAHDM were much more than commercial composite. Biofilm CFU on composite was reduced by 4 logs (*n* = 9, *p* < 0.05). Biofilm metabolic activity and lactic acid were also substantially reduced by nCaF_2_+DMAHDM, compared to commercial control composite (*p* < 0.05). (4) Conclusions: The novel nanocomposite nCaF_2_+DMAHDM achieved strong antibacterial and ion release capabilities, without compromising the mechanical properties. This bioactive nanocomposite is promising to reduce biofilm acid production, inhibit recurrent caries, and increase restoration longevity.

## 1. Introduction

Dental resin composites are an excellent material for direct restorations of anterior teeth and in many cases posterior teeth due to their esthetics and ease of placement [1]. Nevertheless, composites are known to accumulate more oral bacterial plaque and biofilm than other direct restorative materials, which could expose the restored tooth to a higher risk for future recurrent caries [2]. Indeed, most failed restorations due to secondary caries are restored with composites [3,4]. The formation of plaque starts with the salivary-acquired pellicle formation. The glycoprotein found in the acquired pellicle promotes bacterial cell adherence. The microbes in the biofilm then produce acids which lowers the pH and lead to mineral loss over time resulting in dissolution of the tooth structure, the formation of caries, and failure of the restoration [5,6,7]. Unfortunately, currently available commercial composites lack antibacterial properties. Accordingly, efforts were made to overcome the presence of cariogenic bacteria, in an effort to prevent recurrent caries [8].

The incorporation of calcium fluoride nanoparticles (nCaF_2_) into composites has the potential to reduce demineralization [9]. Fluoride (F) ions work by stimulating the remineralization and suppressing the oral microorganisms [10,11]. The presence of F ions in the event of demineralization enhances the precipitation of calcium and phosphate ions and forms fluorapatite [Ca_5_(PO_4_)_3_F] to protect the tooth surface [12,13,14]. Fluoride was also shown to have the advantage of reducing bacterial acid production to reduce recurrent caries.

Designing a composite containing calcium fluoride nanoparticles would enhance the fluorapatite deposition in the affected tooth structure. When the tooth structure is subjected to acidic attack by the cariogenic pathogens, calcium and phosphate ions are lost from enamel. Using remineralization approaches to restore the lost minerals is required to enforce and strengthen the tooth structure. Therefore, the composite with calcium fluoride nanoparticles would enhance remineralization and form fluorapatite that is able to resist future acidic challenges. Several studies have demonstrated the ability of forming fluorapatite using nanotechnology [15,16]. In one study, they manufactured fluorapatite nanoparticles and examined its doping with silver ion nanoparticles and evaluated its physical and antimicrobial effects. The results showed 30% inhibition of bacterial growth after 4 h of incubation while maintaining the natural morphology of fluorapatite [15]. In another study, fluorapatite was incorporated into chitosan scaffolds. Fluorapatite maintained its structure, granted antimicrobial effects, and showed osteoconductive capability [16].

Furthermore, the incorporation of antibacterial agents into composites have also been investigated. Imazato et al. integrated 12-methacryloyloxydodecylpyridinium bromide (MDPB) into composites and showed successful antibacterial effects [17,18,19,20]. The incorporation of quaternary ammonium polyethylenimine (QPEI) into composites also produced a potent and wide-spectrum antimicrobial effect against salivary microorganisms [21]. Antimicrobial peptides (AMPs) were also demonstrated to have antimicrobial properties by bacterial membrane permeabilization and intracellular targeting [22]. Other studies developed antibacterial agents such as dimethylaminohexadecyl methacrylate (DMAHDM) [23,24] and showed a strong antibiofilm activity without compromising the mechanical properties [25].

Previous studies indicated that the salivary protein accumulation on composite surface could lower the efficiency of “contact-killing” mechanisms [26,27]. Accordingly, efforts were made to improve protein-repellent strategies including the addition of protein-repellent agents such as (2-methacryloyloxyethyl phosphorylcholine, or MPC) into resins [28,29,30]. This method provided resistance to protein adsorption and bacterial adhesion due to the hydrophilic characteristic of MPC [29,30]. However, to date, there has been no report on the development of a novel bioactive dental composite that contains nCaF_2_, DMAHDM, and MPC in combination.

The objectives of this study were to develop a new composite consisting nCaF_2_, DMAHDM, and MPC, and to investigate the mechanical, ion release and oral biofilm properties for the first time. The following hypotheses were tested: (1) Adding DMAHDM and MPC into the nCaF_2_ composite would have mechanical properties similar to a commercial control composite; (2) Adding DMAHDM and MPC into the nCaF_2_ composite would not compromise the F and Ca ion release; and (3) The new bioactive composite would have much less microorganisms, produce less biofilm acid, and have better remineralizing properties than the commercial control composite.

## 2. Materials and Methods

### 2.1. Fabrication of Composites

The experimental resin consisted of bisphenol A glycidyl dimethacrylate (BisGMA, Esstech, Essington, PA, USA), and triethylene glycol dimethacrylate (TEGDMA, Esstech) at 50:50 mass ratio. Camphorquinone at 0.2% (Millipore Sigma, Burlington, MA, USA) and 0.8% ethyl 4-N, N-diethylaminobenzoate (Millipore Sigma) were incorporated for photoactivation. The resin is referred to as BT resin. MPC (Millipore Sigma) was added at a mass fraction of 3% and incorporated into the BT resin with magnetic stirring bar at 150 rpm to be dissolved completely into the resin.

The synthesis of DMAHDM was performed using a modified Menschutkin reaction [31]. Briefly, 10 mmol of 2-(dimethylamino) ethyl methacrylate (Millipore Sigma), 10 mmol of 1-bromohexadecane (TCI America, Portland, OR, USA), and 3 g of ethanol were combined in a reaction vessel and then stirred for 24 h at 70 °C. After the evaporation of the solvent and removal of impurities, the DMADHM was collected. DMAHDM was added into the BT resin at a mass fraction of 3% and was stirred using a magnetic stirring bar at 150 rpm until it was completely dissolved into the resin.

The nCaF_2_ was manufactured using a spray-dry method as described in previous studies, yielding a mean particle size of 32 nm [9,32,33,34]. The mass fraction of nCaF_2_ incorporated into BT resin was 15%, based on our preliminary study. A previous study tested different concentrations of nCaF_2_ in composite and, after long-term water-aging, the composite with 20% nCaF_2_ had a flexural strength of 60 MPa [33]. In the present study, 15% nCaF_2_ was integrated into the resin to achieve good mechanical strength. Silanized barium boroaluminosilicate glass particles with a mean size of 1.4 µm (Dentsply Sirona, Milford, DE, USA) were incorporated into the BT resin for mechanical enhancement. As a commercial control composite, Heliomolar (Ivoclar Vivadent, Mississauga, ON, Canada) was also tested. Heliomolar contains 66.7% filler mass fraction of ytterbium-trifluoride and nanofillers of 40–200 nm of silica. The following groups were tested (Table 1 summarizes the materials used in the study):

1. Heliomolar (referred to as commercial control (CC));
2. BT Resin + 70% glass (referred to as experimental control (EC));
3. Remineralizing composite: BT + 15% nCaF_2_ + 55% glass (referred to as nCaF_2_);
4. Antibacterial and remineralizing composite: BT + 15% nCaF_2_ + 3% DMAHDM + 55% glass (referred to as nCaF_2_+DMAHDM);
5. Protein-repellent and remineralizing composite: BT + 15% nCaF_2_ + 3% MPC + 55% glass (referred to as nCaF_2_+MPC);
6. Antibacterial, protein-repellent, and remineralizing composite: BT + 15% nCaF_2_ + 3% DMAHDM + 3% MPC + 55% glass (referred to as nCaF_2_+DMAHDM+MPC).

### 2.2. Characterization of nCaF_2_

Transmission electron microscopy (TEM, Tecnai T12, FEI, Hillsboro, OR, USA) was used to assess the nanoparticles. Samples were prepared through placing nanoparticles on a perforated copper grid coated by a carbon film. To avoid particle agglomeration, the sample was ultrasonicated for 5 min in acetone prior to deposition. Particle size distribution was measured using a laser diffraction particle size analyzer (SALD-2300, Shimadzu North America, Columbia, MD, USA).

### 2.3. Mechanical Properties Testing

Each composite paste was mixed in a disposable plastic container using a speed mixer (DAC 150.1 FVZ-K SpeedMixer™, FlackTec Inc., Landrum, SC, USA) at a speed of 2800 rpm for 1 min, and then thoroughly mixed by hand on a plastic slab for 5 min. The paste was then placed in a rectangular mold of 2 × 2 × 25 mm^3^. Mylar strips were placed on both sides, followed by two glass slides. The specimen was light-cured using a curing unit at 1200 mW/cm^2^ (Labolight DUO, GC America, Alsip, IL, USA) on each side for 1 min [35]. After demolding, the samples were stored in a 100% humidity chamber for 24 h at 37 °C. Flexural strength and elastic modulus were tested at a crosshead-speed of 1 mm/min with a 10 mm span with a three-point flexural test using a computer-controlled universal testing system (Insight 1, MTS, Eden Prairie, MN, USA) [36,37]. Flexural strength and elastic modulus were measured after 24 h of specimen immersion in distilled water at 37 °C. Flexural strength: S = 3P_max_/L(2bh^2^), where P_max_ is the fracture load, L is span, b is sample width and h is thickness. Elastic modulus: E = (P/d) (L^3^/[4bh^3^]), where load P was divided by displacement d which is the slope in the linear elastic region. Six specimens were tested for each group (*n* = 6).

### 2.4. Ca and F Ion Release

The ion releases for all groups containing nCaF_2_ were tested. A solution of sodium chloride (NaCl) (133 mmol/L) was buffered with 50 mmol/L HEPES to pH 7 [36,38]. Three specimens of 2 × 2 × 12 mm^3^ were placed into 50 mL of solution, accommodating a specimen volume/solution ratio of 3.0 mm^3^/mL, similar to those in previous studies [36,38,39]. The specimen’s F and Ca ions release were measured at 1, 2, 4, 7, 14, 21, 28, 35, 42, 49, 56, 63, and 70 days. At every time point, aliquots of 2 mL were collected and substituted by a fresh 2 mL solution of NaCl. The aliquots were investigated for Ca ions by a colorimetric assay using a microplate reader (SpectraMax M5, Molecular Probes, San Jose, CA, USA) as previously described, using known standard and calibration curves [36,38,39]. The F ion release was tested with a F ion selective electrode (Orion, Cambridge, MA, USA). Fluoride standard solutions were measured to form a standard curve. The standard curve was used to establish the F concentration. The F ion concentration measurement was performed by combining 0.5 mL of sample and 0.5 mL of undiluted TISAB solution (Fisher Scientific, Pittsburgh, PA, USA).

### 2.5. Sample Preparation for Biofilm Tests

The cover of a 96 well plate was used to fabricate composite discs for microbiological experiments yielding samples 0.5 mm in thickness and 8 mm in diameter [31]. Composite paste was placed at each indent in the in 96-well plate cover then covered with Mylar strips and glass slides to form a smooth surface. It was then light cured as described previously and then stored for 24 h at 37 °C. The following day discs were magnetically stirred for 1 h at 100 rpm in distilled water to remove uncured monomers [18,40,41]. The specimens were sterilized using ethylene oxide (Anprolene AN 74i, Andersen Products, Haw River, NC, USA) for 24 and allowed to de-gas for 7 days, following the instructions of the manufacturer.

### 2.6. Saliva Collection and Dental Plaque Microcosm Biofilm Model

Saliva collection was conducted in accordance with the Declaration of Helsinki, and the protocol was approved by the Institutional Review Board at the University of Maryland Baltimore (IRB #: HP-00050407). The advantage of the dental plaque microcosm biofilm model is the use of an inoculum of human saliva to mimic the heterogeneity and complexity of the bacteria that are present in human dental plaque [18]. An equal amount of saliva was simultaneously gathered from ten healthy contributors with normal dentition, free of active caries, and no antibiotic use within the prior 3 months. Contributors were instructed not to brush their teeth 24 h preceding collection and not to eat or drink 2 h preceding the collection. Subsequently, the collected saliva from all participants was mixed and diluted to 70% in sterile glycerol. Then the saliva–glycerol solution was stored at −80 °C until use [42].

For all biofilm experiments, McBain artificial saliva growth medium was used. McBain medium contained 2.5 g/L Type II mucin (porcine, gastric, Millipore Sigma), 2.0 g/L bacteriological peptone (Becton Dickinson, Sparks, MD, USA), 2.0 g/L tryptone (Becton Dickinson), 0.35 g/L NaCl, 1.0 g/L yeast extract (Fisher Scientific), 0.2 g/L potassium chloride (Millipore Sigma), 0.1 g/L cysteine hydrochloride (Millipore Sigma), 0.2 g/L calcium chloride (Millipore Sigma). The pH of the medium was adjusted to 7 and autoclaved. After cooling the medium, 0.0002 g/L vitamin K_1_, 0.001 g/L hemin were added. During biofilm experiments, 2% sucrose solution and the saliva–glycerol solution were used as an inoculum at a ratio of 1:50. The sucrose and inoculum were added to the medium and 1.5 mL of the medium was placed in each well of a 24-well plate containing a composite specimen from each groups. Specimen were incubated in 5% CO_2_ at 37 °C for 8 h to permit biofilm growth on the samples. The same procedure was repeated after 8 h without the addition of saliva and incubation occurred again for 16 h. After 16 h the samples were moved to a new 24-well plate which contained fresh medium and sucrose, and was further incubated for 24 h. Composites were exposed to bacterial culture for a total of 48 h, which resulted in reasonably mature dental plaque microcosm biofilms on composites [25,43].

### 2.7. Biofilm Colony Forming Units (CFU) Counts

Nine discs were prepared for each group. Following the 48 h incubation, the disc samples containing biofilm were transported into a vial filled with 1 mL of cysteine peptone water (CPW). This was vortexed for 5 s then sonicated for 5 min and vortexed again to harvest the biofilm [29]. Serial dilutions of the suspensions of bacteria were prepared and transported to agar plates to grow. The CFU were counted on three different agar plates. To determine total streptococci count, mitis salivarius agar (MSA, Becton Dickinson, Sparks, MD, USA) were used. To determine the growth of mutans streptococci, 0.2 units per mL bacitracin (Millipore Sigma) was added to the mitis salivarius agar (MSB). To evaluate the growth of the total microorganisms, tryptic soy blood agar (TSBA) agar plates were used by adding defibrinated sheep blood to tryptic soy agar (TSA, Becton Dickinson). The agar plates were kept at 37 °C in a 5% CO_2_ incubator for 48 h. CFU calculation was based on the colony number and multiplied by the dilution factor [29].

### 2.8. Biofilms Metabolic Activity Evaluation (MTT)

The MTT (3-[4,5-dimethylthiazol-2-yl]-2,5- diphenyltetrazolium bromide) assay was performed to investigate the biofilm metabolic activity. Following 48 h of incubation, the discs (*n* = 9) were transferred into a clean 24-well plate, then 1 mL of tetrazolium dye was placed to every disc. The discs were then incubated in an incubator of 5% CO_2_ at 37 °C. Discs were then transported to another 24-well plate, and 1 mL of dimethyl sulfoxide (DMSO) was added to every disc and incubated for 20 min in a dark room [29,44]. After incubation, 200 µL of the DMSO solution was collected and the absorbance at 540 nm was measured [29,44] using a microplate reader (SpectraMax^®^ M5, Molecular Devices, San Jose, CA, USA).

### 2.9. Biofilms Lactic Acid Production

Following 48 h incubation, discs (*n* = 9) were moved to a different 24-well plate comprising 1.5 mL buffered-peptone water (BPW) with 0.2% sucrose then incubated for 3 h in a 5% CO_2_ incubator at 37 °C to release acids. After 3 h, the BPW solution lactic acid concentrations were measured by recording the absorbance at 340 nm [29,44] using a microplate reader (SpectraMax^®^ M5, Molecular Devices). Standard curves were produced by means of lactic acid standards.

### 2.10. Scanning Electron Microscopy (SEM) of Biofilms

For biofilm visualization and confirmation of bacterial attachments on composite discs, biofilms formed at 24 h, 48 h, and 96 h were sputter-coated with platinum. Scanning electron microscopy (SEM, Quanta 200, FEI Company, Hillsboro, OR, USA) was used to examine the bacterial accumulation (Figure 7).

## 3. Statistical Analysis

All data were evaluated with one-way analysis of variance (ANOVA), and post hoc multiple comparison using Tukey’s honestly significant difference test was performed. All statistical analysis was completed using the GraphPad Prism 8 software package (GraphPad Software, San Diego, CA, USA) at 0.05 level of significance.

## 4. Results

**Figure 1:**
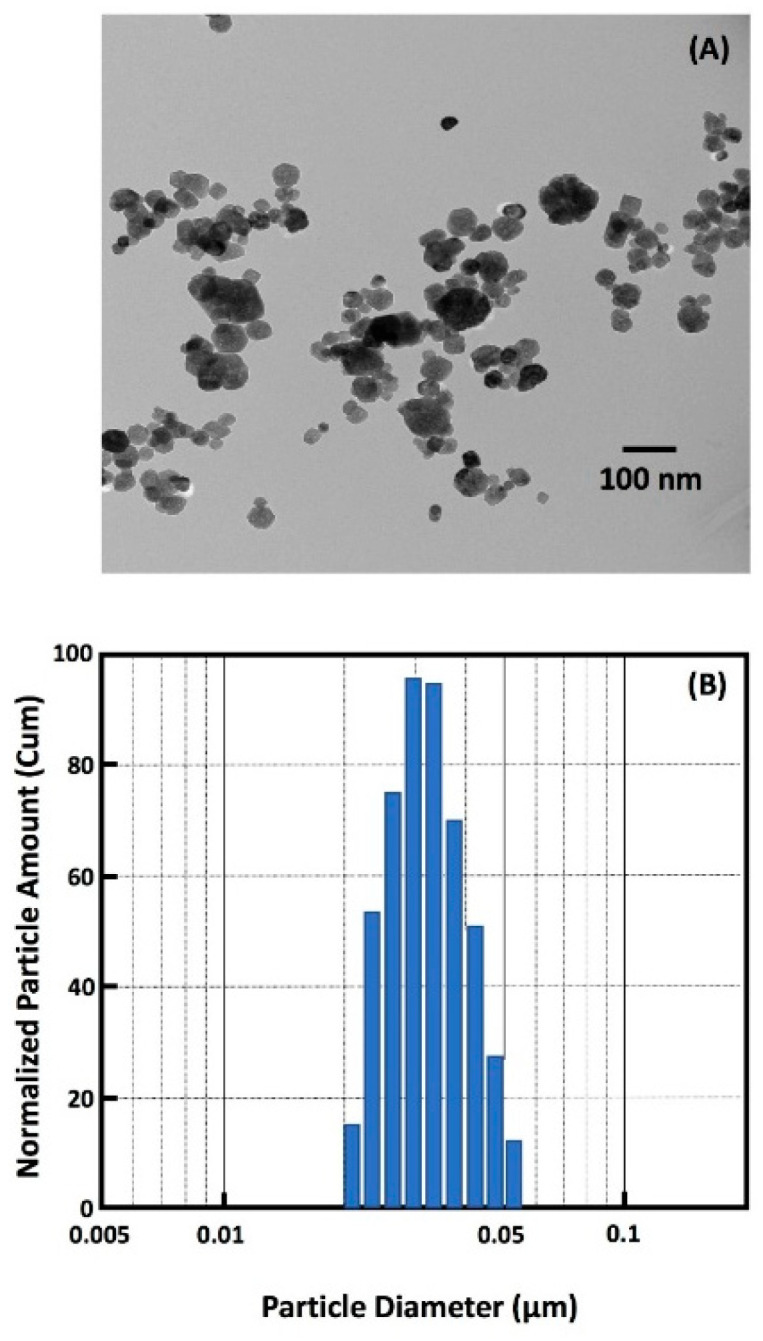
(**A**) TEM of nanoparticles of CaF_2_ (nCaF_2_) synthesized in this study. (**B**) Particle size distribution of nCaF_2_. The nCaF_2_ were synthesized via a spray-drying technique and collected using an electrostatic precipitator.

A representative TEM image of calcium fluoride (nCaF_2_) nanoparticles is shown in Figure 1A. The nanoparticle size distribution ranged from 22 nm to 57 nm, with a mean particle size of 32 nm and is illustrated in Figure 1B.

**Figure 2:**
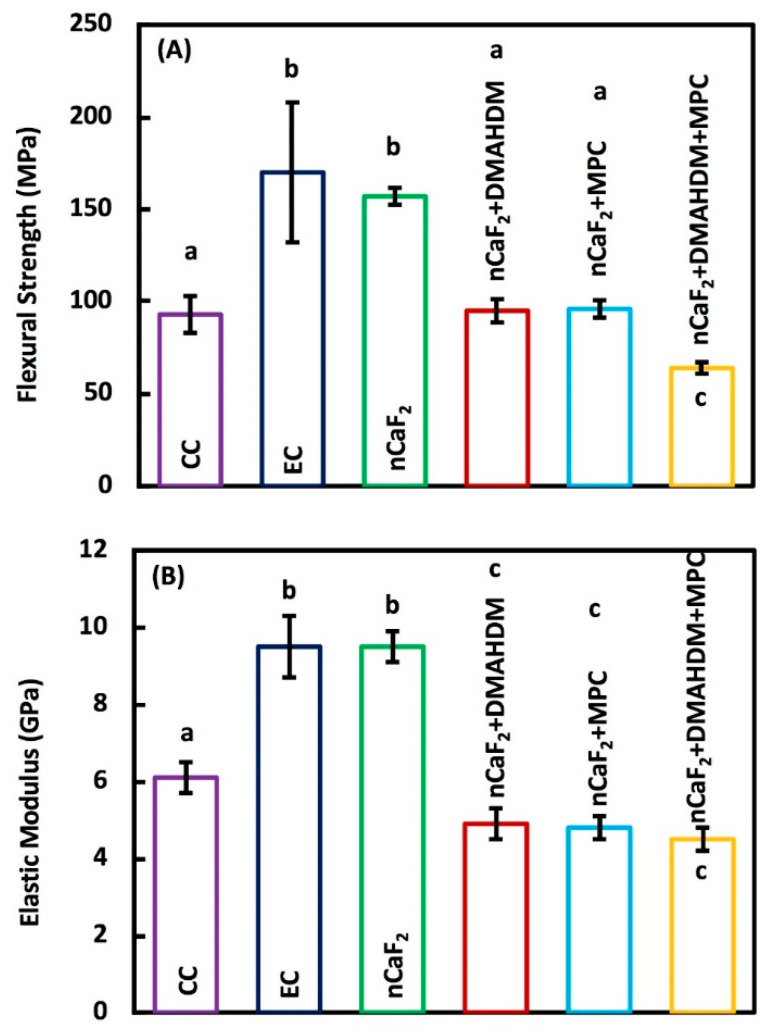
Mechanical properties of composites: (**A**) Flexural strength, and (**B**) Elastic modulus (mean ± sd; *n* = 6). The flexural strength was higher in experimental control (EC) and nCaF_2_ than commercial Heliomolar control (*p* < 0.05). Flexural strength in nCaF_2_+DMAHDM and nCaF_2_+MPC matched Heliomolar control (*p* > 0.05). However, the flexural strength in nCaF_2_+DMAHDM+MPC was reduced (*p* < 0.05). The elastic modulus of the EC and nCaF_2_ were higher than Heliomolar control (*p* < 0.05). All other groups had comparable elastic moduli to Heliomolar control after 1 day of immersion (*p* > 0.05). In each plot, different letters (a, b, c) indicate values that are significantly different from each other (*p* < 0.05).

Flexural strength and elastic modulus of the six composite groups (mean ± sd; *n* = 6) are shown in Figure 2A,B, respectively. The flexural strength was measured after one day of immersion in water at 37 °C. The flexural strength was significantly higher in EC and nCaF_2_ composites, when compared to the commercial Heliomolar control (*p* < 0.05). Flexural strength in groups with nCaF_2_+DMAHDM and nCaF_2_+MPC matched those of Heliomolar control composite (*p* > 0.05). However, flexural strength in nCaF_2_+DMAHDM+MPC was significantly lower than commercial Heliomolar control (*p* < 0.05).

The elastic modulus values of EC and nCaF_2_ were significantly greater than all other groups (*p* < 0.05). Other groups had comparable elastic modulus values to Heliomolar control (*p* < 0.05).

**Figure 3:**
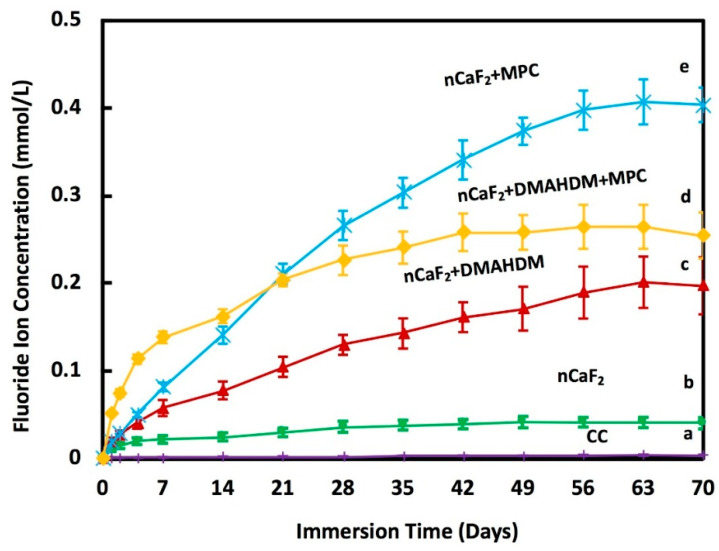
Fluoride (F) ion release from composites (mean ± sd; *n* = 6) at pH 7.0. The incorporation of nCaF_2_, DMAHDM, and MPC increased the release of F ions with time (*p* < 0.05). Different letters (a, b, c, d, e) indicate significant differences between groups at day 70 (*p* < 0.05).

The accumulative F ion release is shown in Figure 3. At 70 days, the composite nCaF_2_+MPC had the highest F release of (0.40 ± 0.02) mmol/L (*p* < 0.05). Meanwhile, nCaF_2_+DMAHDM+MPC had F release of (0.25 ± 0.03) mmol/L, nCaF_2_+DMAHDM had (0.20 ± 0.03) mmol/L, and nCaF_2_ had (0.04 ± 0.01) mmol/L of fluoride ion release. Heliomolar control had the lowest F release of (0.004 ± 0.0003) mmol/L.

**Figure 4:**
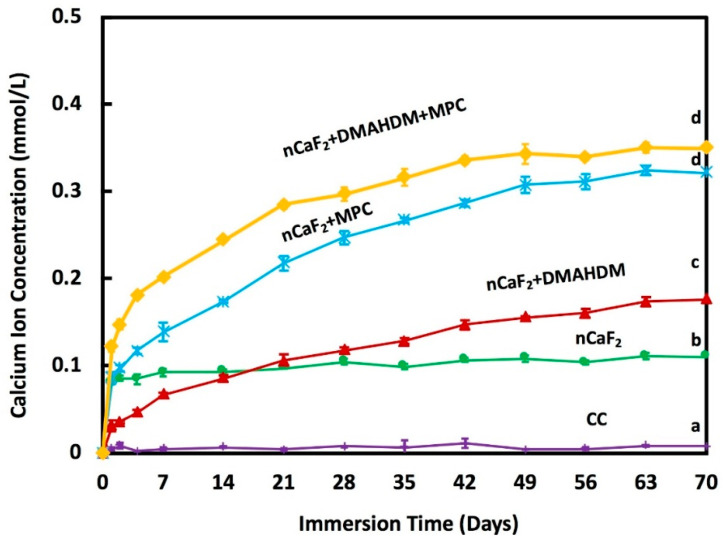
Calcium (Ca) ion release from composite resins (mean ± sd; *n* = 6) at pH 7.0. The incorporation of nCaF_2_, DMAHDM, and MPC increased the release of Ca ions with time (*p* < 0.05). Different letters (a, b, c, d) indicate significant differences between groups at day 70 (*p* < 0.05).

The calcium ion release is plotted in Figure 4. At 70 days, nCaF_2_+MPC had a Ca ion release of (0.32 ± 0.005) mmol/L, and nCaF_2_+DMAHDM+MPC had a similar ion release at (0.35 ± 0.006) mmol/L. Groups containing MPC had ion releases that were significantly higher when compared to other groups (*p* < 0.05). nCaF_2_ had a Ca ion release of (0.11 ± 0.004) mmol/L. nCaF_2_+DMAHDM had (0.18 ± 0.005) mmol/L, and Heliomolar control had close to zero Ca ion release.

**Figure 5:**
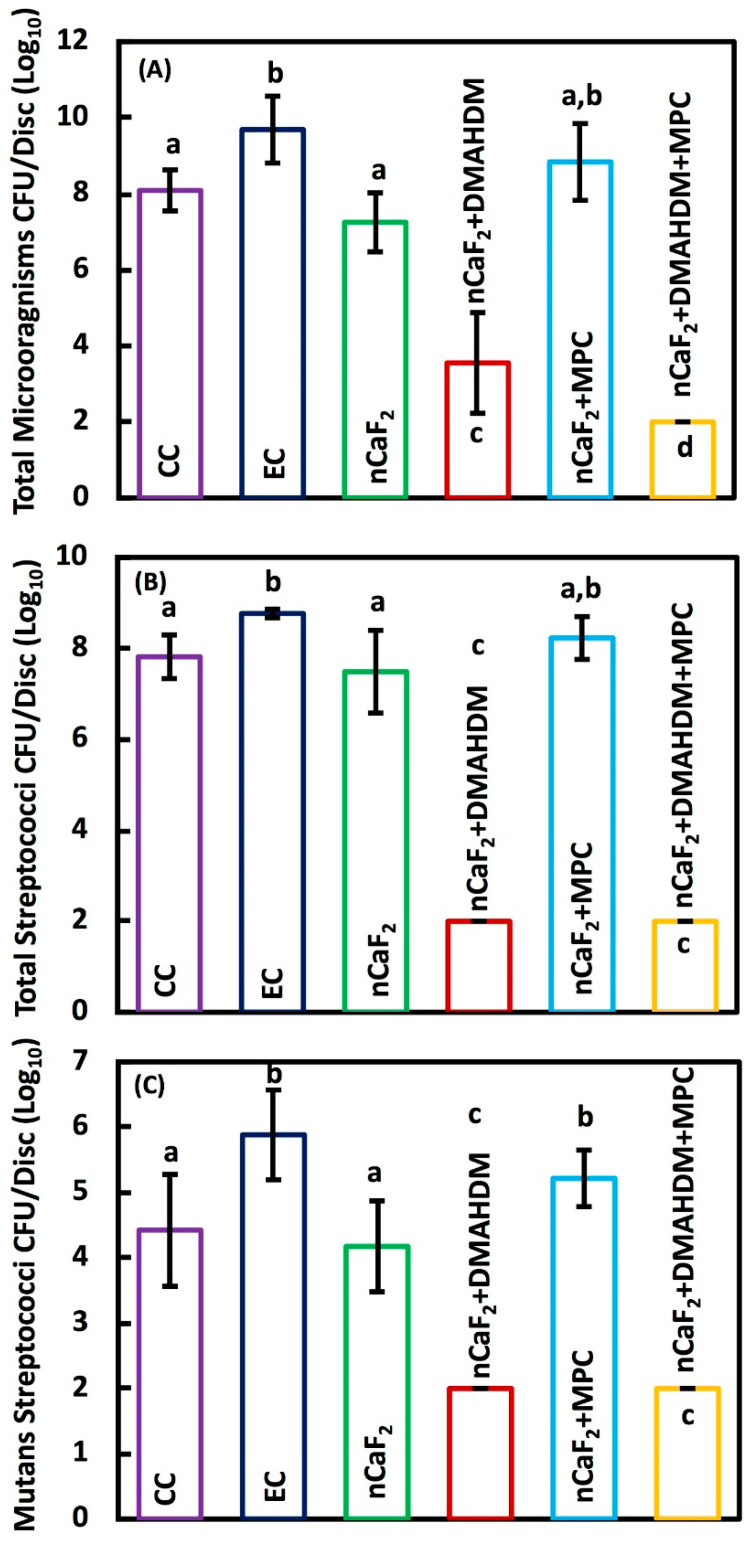
Colony-forming unit (CFU) counts of 2-day biofilm on composite discs (mean ± sd; *n* = 9): (**A**) Total microorganisms, (**B**) total streptococci, and (**C**) mutans streptococci (mean ± sd; *n* = 9).The incorporation of nCaF_2_+DMAHDM+MPC had the lowest CFU, followed by nCaF_2_+DMAHDM in the total microorganisms. The reduction of CFU in nCaF_2_+DMAHDM+MPC and nCaF_2_+DMAHDM was similar in total streptococci and mutans streptococci (*p* < 0.05). In each plot, different letters (a, b, c, d) indicate significant differences between groups (*p* < 0.05).

Two-day biofilm colony forming units CFU on composites are shown in Figure 5: (A) Total microorganisms, (B) total streptococci and (C) mutans streptococci (mean ± SD; *n* = 9). CFU was reduced by 6 logs from a mean of 2.51 × 10^8^ counts for Heliomolar control to 1.00 × 10^2^ counts for the new nCaF_2_+DMAHDM+MPC composite (*p* < 0.05). nCaF_2_+DMAHDM reduced the CFU by 4 logs (*p* < 0.05). The combination of nCaF_2_+DMAHDM+MPC yielded the smallest CFU counts.

**Figure 6:**
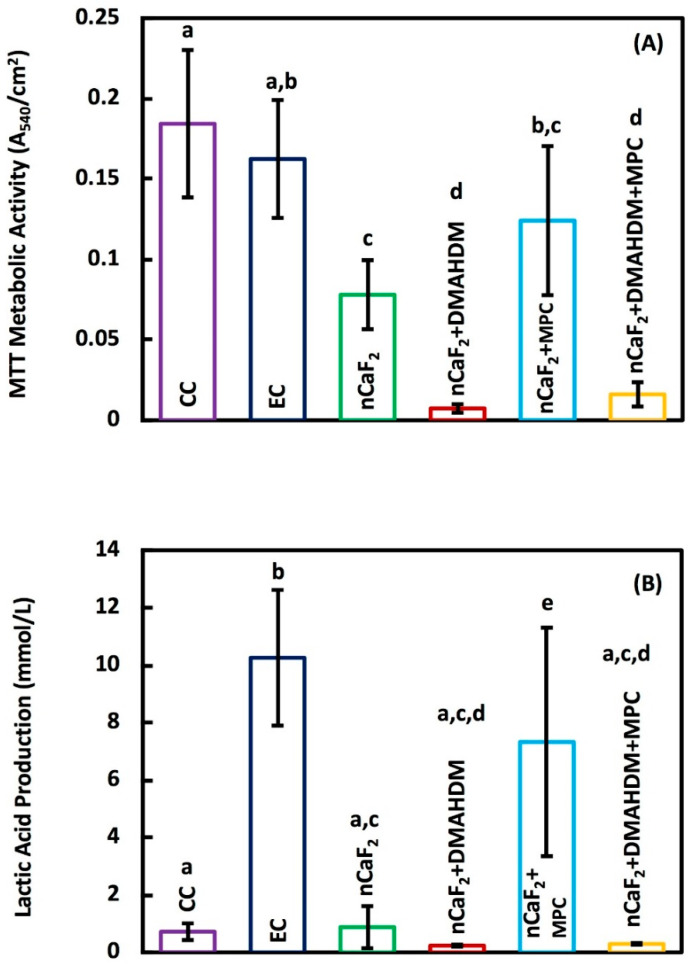
(**A**) The total metabolic activity, and (**B**) Lactic acid production (mean ± sd; *n* = 9) of composite specimens exposed to salivary biofilm. The incorporation of nCaF_2_+ DMAHDM had the best reduction in the metabolic activity and lactic acid production (*p* < 0.05). In each plot, different letters (a, b, c, d) indicate significant differences between groups (*p* < 0.05).

The bacterial metabolic activity of 2 days biofilm on composites is shown in Figure 6A. Metabolic activity was decreased from 0.18 (OD_540_/cm^2^) for commercial Heliomolar composite control, to 0.02 for the nCaF_2_+DMAHDM+MPC composite (*p* < 0.05). Biofilm lactic acid production results can be seen in Figure 6B. The lactic acid production was reduced from 0.72 mmol/L on commercial Heliomolar control composite to 0.29 mmol/L in the nCaF_2_+DMAHDM+MPC composite.

**Figure 7:**
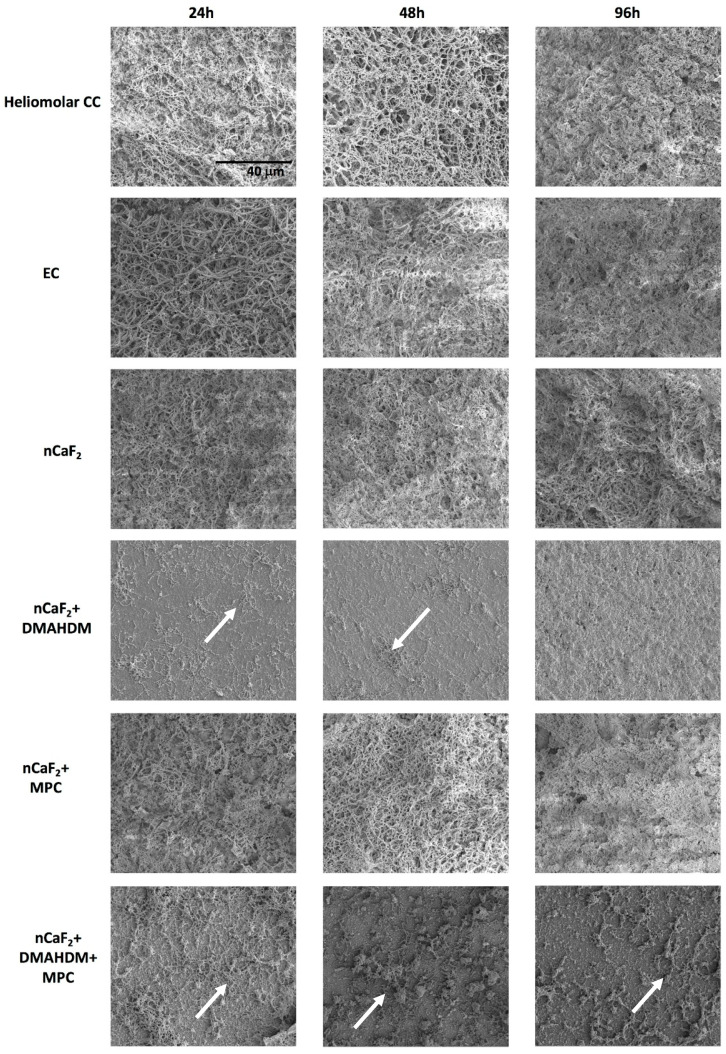
Scanning Electron Microscopy (SEM) images of biofilm formed on composite disc surfaces at 24 h, 48 h, and 96 h. All images are at 3000× magnification. Arrows indicate presence of minimal biofilm on DMAHDM-containing specimens. All other groups had full coverage of a substantial and mature biofilm.

SEM results in Figure 7 indicate an extensive biofilm formation at 24 h, 48 h, and 96 h in all groups except those containing the antimicrobial DMAHDM. Heliomolar (CC), experimental control (EC), and nCaF_2_+MPC groups had the most biofilm formation at all time points, followed by the nCaF_2_ group. Biofilm formation increased with time. However, in DMAHDM-containing groups there was minimal attachment of biofilm observed. At 96 h, the DMAHDM-containing groups showed a reduction in biofilm attachment compared with earlier time points.

## 5. Discussion

The present study developed a new composite consisting of nCaF_2_, DMAHDM and MPC, and investigated the mechanical, ion release and oral biofilm properties. The protein-repellant nCaF_2_+DMAHDM+MPC had slightly lower mechanical properties when compared to all other groups. However, nCaF_2_+DMAHDM had good mechanical properties matching those of a commercial composite, while possessing high levels of F and Ca ion release and a strong antibacterial effect. The new bioactive composite nCaF_2_+DMAHDM decreased biofilm CFU by four orders of magnitude over that of commercial control composite, and substantially reduced acid production to inhibit caries.

Critical to the development of any new composite restorative material are its mechanical properties. The flexural strength of EC was around (170 ± 38) MPa. With the addition of 15% nCaF_2_, the strength was reduced slightly to (157 ± 5) MPa. Those values are consistent with the results in a previous study [32]. The composite in that study had 65% total fillers and 20% CaF_2_, similar to the 70% total fillers and 15% nCaF_2_ composition in the present study. The flexural strength for the control group containing no nCaF_2_ in the previous study was (145 ± 9) MPa, while the group which had 20% nCaF_2_ had a flexural strength of 121 MPa. The nCaF_2_+DMAHDM and nCaF_2_+MPC had similar strengths of (95 ± 6), and (96 ± 5) MPa, respectively. Meanwhile, for nCaF_2_+DMAHDM+MPC, the flexural strength was around (64 ±3) MPa. Those are similar to the results of a previous study using the same filler mass fraction where the flexural strength was around 90 MPa when 3% MPC and 3% DMAHDM were added to the BT resin [43]. The decreased strength in nCaF_2_+DMAHDM+MPC could be due to two reasons. First, the BT base resin is composed of BisGMA and TEGDMA, which contain two reactive groups each. However, both DMAHDM and MPC are monomethacrylates and contain one reactive group. The addition of these monomethacrylates may substantially change the reaction kinetics and resulting crosslinked network, which can result in inferior mechanical properties. Another possible explanation for the decrease in mechanical properties is due to the hydrophilic nature of MPC. The specimens for flexural strength were stored for 24 h in water at 37 °C. The hydrophilicity of MPC makes it more likely that the composite would absorb water during the 24 h immersion prior to testing. This would likely lead to the reduction in flexural strength compared with the non-MPC groups.

Fluoride releasing materials have been known to render teeth more resistant to decay [45]. However, many available materials with fluoride-releasing properties, such as glass ionomer and resin modified glass ionomers, have inferior mechanical properties that do not serve the high load requirements for restorations in stress-bearing areas. Therefore, the incorporation of F ions into composite restorations would have the dual benefit of fluoride release and improved mechanical properties [46,47,48]. The current study synthesized nCaF_2_ through a spray-drying method, which produced nanoparticles of CaF_2_ with a median particle size of about 32 nm. The use of small amounts of nCaF_2_ fillers prevented the mechanical properties to be compromised. As seen in previous studies, the use of fluoride fillers with larger particle sizes require higher mass fractions of fillers to achieve significant fluoride ion release. This would lead to a decrease in the composite mechanical properties [49].

The incorporation of nanoparticles with a high surface area resulted in the release of high levels of F and Ca ions using a low nCaF_2_ filler level [33]. F ions foster remineralization by forming fluoroapatite [Ca_5_(PO_4_)_3_F] [50]. Furthermore, it was shown that the lower the pH, the higher the release of ions. In this study, the release of F ions was significantly increased with the integration of 15% nCaF_2_ at a pH of 7, measuring (0.04 ± 0.01) mmol/L. This was comparable to the 0.03 mmol/L F release level in a study that had a pH of 6, yet with an increased percentage of CaF_2_ (23%) [51]. Groups containing MPC had the highest F ion release over time. The levels of release in nCaF_2_+MPC and nCaF_2_+DMAHDM+MPC were (0.40 ± 0.02) mmol/L and (0.25 ± 0.03) mmol/L, respectively. This could be due to the hydrophilic nature of MPC, resulting in a higher level of water uptake. As more water was absorbed by the composite, the Ca and F ions were solubilized, leading to an increase in the initial ion release. However, nCaF_2_+DMAHDM F ion release was (0.20 ± 0.03) mmol/L, which was superior to the control. While the amount of nCaF_2_ was the same in the two groups, the lower level of initial release in nCaF_2_+DMAHDM, when compared to MPC containing groups, could result in a more sustained level of F ion release over a longer period. The releasing trend of F and Ca ions were the same for all groups. The groups containing nCaF_2_ had a high F release initially, and subsequently were followed by a steady state release. The higher release of Ca and F ions in all nCaF_2_ composites revealed that CaF_2_ nanoparticles were fast released from the nCaF_2_ composites because of the hydrolytic breakdown of the interface between the resin matrix and the CaF_2_ nanoparticles. A likely explanation for such a trend is that the ions were released from the near-surface Ca and F reservoir of the nCaF_2_ composite, and the reservoir near the surface diminished with the increase in immersion time. However, in clinical situations, the superficial layer of nCaF_2_ would experience repeated chewing forces and tooth-brushing, hence the CaF_2_-depleted surface would be removed by wear. As a result, a fresh surface would be exposed with the ability to further continue the calcium and fluoride release. Further studies are needed to investigate the outcome of wear on Ca and F ion release, as well as whether these materials can be made rechargeable to extend the lifetime of ion release.

Recurrent caries is considered a major drawback of composite restorations [1,52,53]. Studies proposed that the greater vulnerability to recurrent caries may be associated with the absence of composites’ antibacterial capability when compared to other commonly used restorative materials such as glass ionomers and amalgams [2,54]. Therefore, the improvement of composites by incorporating antibacterial, protein repellent, and remineralizing properties is essential to lengthen the service life of composite restorations. DMAHDM has demonstrated strong antibacterial effects on an extensive antimicrobial spectrum through its contact-killing properties [26,55]. Since DMAHDM contains a reactive methacrylate group, it is copolymerized within the resin matrix, rendering it immobilized and preventing its release or loss over time [26,56]. Hence, its antibacterial capability is long-lasting. However, the antibacterial ability of DMAHDM is limited to biofilms that contact the composite surface [26], as biofilms that are not in contact with DMAHDM cannot be killed via DMAHDM’s mechanism of action. Even with this limitation, the composite containing nCaF_2_+DMAHDM substantially lowered the biofilm development and production of lactic acid, reducing the CFU of biofilm by 4 logs.

It can be seen in Figure 6B that the experimental control group and the group containing nCaF_2_ and MPC exhibited a greater lactic acid production when compared with the other groups. DMAHDM has been shown in previous work to have a strong antibacterial effect, as illustrated by the CFU data in Figure 5. This antibacterial activity is reflected indirectly by the lactic acid production. When an *S mutans* biofilm accumulates, the secretion of lactic acid occurs. In the absence of a viable biofilm, the concentration of lactic acid is expected to be very low, as is the case in the DMAHDM-containing groups. Interestingly, the nCaF_2_ and nCaF_2_+MPC groups did not have a significant reduction in CFU (Figure 5), but the nCaF_2_ group showed a significant reduction in lactic acid production in contrast to the nCaF_2_+MPC group. Previously, it has been shown that the inclusion of CaF_2_ in a composite formulation has a moderating effect on the lactic acid production [57]. It has been speculated that the F ion release helped reduce the acid production of the bacteria via the inhibition of metabolic pathways such as the fermentation pathway for lactic acid production, biofilm plaque, and hydrodynamic effects on mass transfer, fluoride delivery, and caries [58]. However, when MPC was added to the composite containing nCaF_2_, there was no decrease in lactic acid production. This response may be related to the interactions between the calcium and fluoride ions and the phosphorylcholine fragment of MPC. The protein-repellent nature of MPC from the hydrogen bond network of water molecules surrounding the phosphorylcholine fragment limited the ability of the protein to adsorb on the surface. It was found, however, that the presence of halide ions (Cl^−^, Br^−^, I^−^) influenced the hydrogen bond network and the diffusion through it [59]. It is possible that the presence of F^−^ ions would have a similar result, which may affect F ion diffusion and disrupt the creation of a protein-repellant coating, leading to biofilm formation.

SEM images in Figure 7 confirm the presence of substantial biofilm on all groups except those containing DMAHDM. At 24 h and 48 h, Heliomolar (CC), experimental control (EC), nCaF_2_, and nCaF_2_+MPC groups exhibited the formation of microcolonies and early biofilm multilayers. The biofilms on these groups at 96 h were fully mature, substantial, and dense. However, in DMAHDM-containing groups there was minimal attachment of biofilm observed. At 96 h the DMAHDM-containing groups showed a reduction in biofilm attachment compared to earlier time points. It is possible that the antimicrobial effect of DMAHDM as a contact-killing agent had eliminated the bacteria left on the samples over time. These results correlate very well with the CFU results.

It is possible that the salivary proteins adsorption on composite resin surfaces might reduce the effectiveness of this “contact-killing” mechanism. Therefore, a protein repellent agent was added. MPC is a methacrylate that contains phospholipid polar groups. MPC is highly hydrophilic and has been shown to decrease the adsorption of protein and reduce bacterial attachments [60]. Previous work indicated that integrating MPC into the resin decreased the adsorption of proteins by approximately one order of magnitude [43,61]. Additionally, it was shown that surfaces containing MPC were resistant to brushing mechanical stresses [62]. In this study, MPC was incorporated into the resin, and copolymerized into the composite resin. This is analogous to the process of incorporating DMAHDM, with a similar result of a stable, covalently bonded, and non-releasing functionality.

In the current study, the antibacterial capability of the experimental composite was improved with nCaF_2_ and was further substantially improved with the use of both nCaF_2_ and DMAHDM. However, the nCaF_2_+MPC group showed no reduction in the CFU counts. The composite that had nCaF_2_+DMAHDM+MPC displayed the most potent antibacterial effect with CFU 6 log biofilm reduction. Therefore, these results confirmed that the addition of DMAHDM is vital to improving the antibacterial effect. It is thought that DMAHDM serves to reduce the accumulation of biofilm and, as a result, improves the efficacy of MPC in resisting protein adsorption. However, in the absence of DMAHDM, the bacterial biofilm will accumulate and render MPC less effective.

The combination of nCaF_2_+DMAHDM promoted remineralization and had a significant antibacterial effect. Another potential benefit of using nCaF_2_ is that the release of F ions could act as a transmembrane proton carrier and an inhibitor to the glycolytic enzyme, thus preventing oral microorganisms by stimulating acidification of the cytoplasm and providing antibacterial effect at a long-distance to inhibit caries [10,63]. These properties would be highly valuable to inhibit recurrent caries around the margins of the restoration, since this is the location that most dental plaque tends to accumulate. In addition to its superior antibacterial and remineralization properties, the nCaF_2_+DMAHDM composite exhibits excellent mechanical properties which make it suitable in a variety of restoration applications. In comparison, the composite consisting of nCaF_2_+DMAHDM+MPC exhibited lower mechanical properties. However, the strength and modulus achieved by this group was still high enough to be used for restorations in low load areas such as class V restorations [37]. This is a critical area of utilization, since older patients tend to have exposed root surfaces that are more prone to caries. Further studies are needed to investigate the nCaF_2_+DMAHDM combination in dental cements, bonding agents, fissure sealants, and composites to remineralize tooth lesions and suppress biofilm and plaque buildup, especially in patients with a high caries risk.

## 6. Conclusions

A novel composite with remineralization and antibacterial properties was established by combining nCaF_2_+DMAHDM for the first time. Release of F and Ca ions from the composite was significantly achieved through the incorporation of nCaF_2_. The new composite exhibited strong antibacterial and ion release capabilities, prevented biofilm production of lactic acid, and decreased biofilm CFU by 4 log. This bioactive nanocomposite is promising to protect tooth structures, inhibit demineralization, and provide a reservoir for the release of calcium and fluoride ions. The combined effect of nCaF_2_+DMAHDM shows promise in a variety of dental applications where remineralization and prevention of recurrent caries is a priority.

## Figures and Tables

**Table 1:** Materials used in the study.

Formulation/Manufacturer	Abbreviation	Fluoride	DMAHDM	MPC
Heliomolar, Ivoclar Vivadent, Mississauga, ON, Canada (Commercial control)	CC	**+**	**-**	**-**
30% BT+70% Glass (Experimental Control)	EC	**-**	**-**	**-**
30% BT+15% nCaF_2_+55% Glass	nCaF_2_	**+**	**-**	**-**
27% BT+15% nCaF_2_+3%DMAHDM +55% Glass	nCaF_2_+ DMAHDM	**+**	**+**	**-**
27% BT+15% nCaF_2_+3% MPC +55% Glass	nCaF_2_+MPC	**+**	**-**	**+**
24% BT+15% nCaF_2_+3% DMAHDM +3% MPC+55% Glass	nCaF_2_+DMAHDM+MPC	**+**	**+**	**+**
